# Predicting acute kidney injury following open partial nephrectomy treatment using SAT-pruned explainable machine learning model

**DOI:** 10.1186/s12911-022-01877-8

**Published:** 2022-05-16

**Authors:** Teddy Lazebnik, Zaher Bahouth, Svetlana Bunimovich-Mendrazitsky, Sarel Halachmi

**Affiliations:** 1 Department of Cancer Biology, Cancer Institute, University College London, London, UK; 2 Department of Urology, Bnai Zion Medical Center, Haifa, Israel; 3 Department of Mathematics, Ariel University, Ariel, Israel

**Keywords:** AKI prediction, SAT pruned random forest, PN treatment complication prediction

## Abstract

**Background:**

One of the most prevalent complications of Partial Nephrectomy (PN) is Acute Kidney Injury (AKI), which could have a negative impact on subsequent renal function and occurs in up to 24.3% of patients undergoing PN. The aim of this study was to predict the occurrence of AKI following PN using preoperative parameters by applying machine learning algorithms.

**Methods:**

We included all adult patients (n = 723) who underwent open PN in our department since 1995 and on whom we have data on the pre-operative renal function. We developed a random forest (RF) model with Boolean satisfaction-based pruned decision trees for binary classification (AKI or non-AKI). Hyper-parameter grid search was performed to optimize the model's performance. Fivefold cross-validation was applied to evaluate the model. We implemented a RF model with greedy feature selection to binary classify AKI and non-AKI cases based on pre-operative data.

**Results:**

The best model obtained a 0.69 precision and 0.69 recall in classifying the AKI and non-AKI groups on average (k = 5). In addition, the model's probability to correctly classify a new prediction is 0.75. The proposed model is available as an online calculator.

**Conclusions:**

Our model predicts the occurrence of AKI following open PN with (75%) accuracy. We plan to externally validate this model and modify it to minimally-invasive PN.

**Supplementary Information:**

The online version contains supplementary material available at 10.1186/s12911-022-01877-8.

## Background

Renal cell carcinoma (RCC) represents about 3% of all cancer-related cases in 2018, with the highest incidence occurring in Western countries [1]. During the last decades, stage migration towards localized disease has occurred [2]. Partial nephrectomy (PN) is the treatment of choice for localized cT1 renal masses [3]. The main advantage of PN is the preservation of renal function compared to radical nephrectomy [4]. One of the adverse effects of PN is post-operative acute kidney injury (AKI), which increases the risk of long-term chronic kidney disease (CKD) with its consequences, including decreased overall survival [5], although some studies questioned its impact on long-term renal function [6]. The prevalence of AKI following PN is reported to be up around 25% and is dependent on the surgical approach, patient baseline characteristics, and the definition of AKI used in each study [7].

Machine learning (ML) based models which predict different clinical properties have been shown to be a useful tool [8] and particularly in clinical practice [9]. ML models can be classified into three main subtypes: classification, search, and prediction. In this paper, we focus on the latter in order to predict AKI following PN.

Prediction ML models provided with retrospective data are able to find complex statistical connections between different parameters (this step is usually referred to as the learning process) [8]. As a result, upon providing a new set of parameters, these models are able to predict, with fair accuracy, the outcome one wishes to retrieve. Weng et al. [10] used four ML algorithms to predict cardiovascular risk, showing improvement in all four compared to standard algorithms. A study by Wu et al. [11] developed an ML model to predict fatty liver disease. The authors used five different ML algorithms on the same data, where RF showed the best results. Several authors used different ML models to predict medical AKI in hospitalized patients [12, 13]. For instance, Kate el al. presented a framework in which AKI is continually predicted automatically from EHR data over the entire hospital stay using [14]. In addition, Gameiro et al. reviewed multiple artificial intelligence based models for AKI risk prediction [15]. The authors concluded that real-time implementation of ML-based AKI risk models is a promising approach as these do not require additional AKI biomarker testing.

Specifically, previous studies investigating the performance of ML models in predicting AKI have yielded promising results [16, 17]. However, the accuracy of these models is not optimal, and we thought that by using an ML model, we can increase the accuracy of these models.

**Fig. 1:**
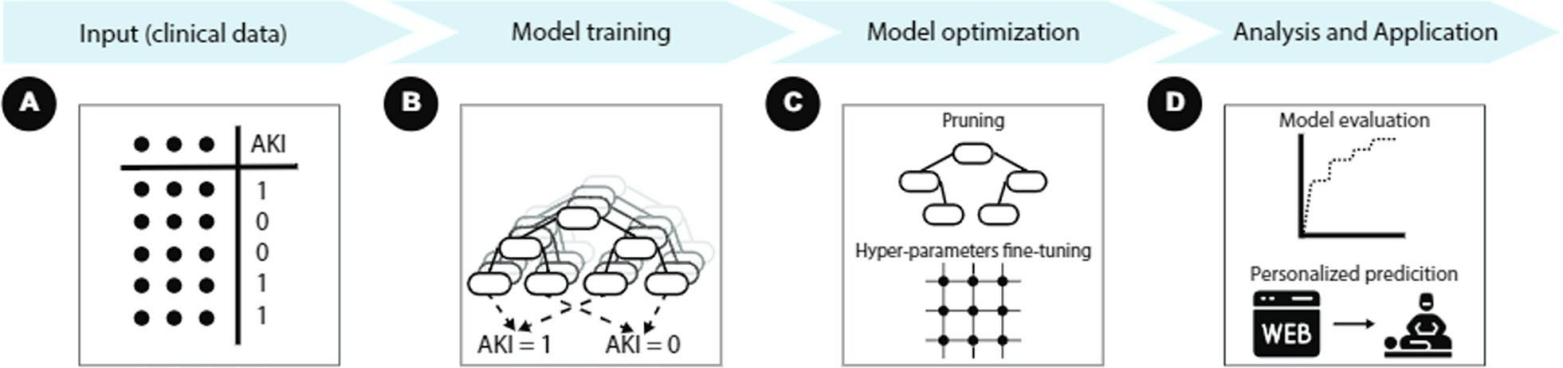
A workflow of the proposed framework

In this study, we aim to apply an explainable ML model to predict AKI in patients undergoing open PN. We hypothesized that ML models could identify and learn from pre-operative parameters and predict the AKI outcome. A self-explainable prediction system that is based on ML was then built and deployed online. A schematic view of the workflow of the proposed framework is shown in Fig. 1.

## Methods

### Data acquisition

Since 1995, we have been continuously extending our open PN database to include surgical and oncological parameters. For this particular study, we included all adult (> 18 years) patients who underwent open PN for enhancing solid renal mass and then split the data into AKI and non-AKI. Patients with a solitary kidney or multiple tumors were excluded from this study. Therefore, the PN database includes 723 patients. Renal function was assessed the day before surgery, on the day of the surgery, and on a daily basis after the surgery until discharge which more often than not was on post-operative day 3.

### Operative technique

An extraperitoneal, extrapleural supra-11th rib incision was done on the operated side. IV Mannitol was given before clamping the renal artery. In situ renal hypothermia was done by cooling the surface of the kidney with ice slush for 10–15 min immediately after clamping the renal artery. The tumor was enucleated with a minimal rim of normal parenchyma. Renorrhaphy was done using either 2/0 VICRYL interrupted sutures or tissue adhesive BioGlue (CryoLife, Atlanta, GA). A more detailed surgical technique has been previously published by our group [18].

### Renal function assessment

Baseline serum Creatinine (sCr) was measured the day before surgery. We used both the RIFLE (risk, injury, failure, loss of kidney function, and end-stage renal failure) [19] and AKIN (Acute Kidney Injury Network) [20] criteria to define AKI, comparing each of the post-operative renal function assessments to the baseline level. AKI was defined as the occurrence of one of the following conditions: (1) an increase in serum Creatinine of ≥ 0.5 times above baseline in the first week following surgery, (2) an increase in sCr by ≥ 0.3 mg/dl(≥ 26.5 mmol/l) above baseline in the 48 h window post-operatively, or (3) reduction of more than 25 percent of the estimated Glomerular Filtration Rate in the 7 days period after surgery. In total, 231 patients developed AKI based on the aforementioned criteria and constituted the AKI group, and 492 did not develop AKI and therefore were classified as non-AKI. 723 patients is considered a large enough set to use for the methods shown in the following sections [21].

### Data split

In order to develop ML algorithm, the study population was compiled into a data set, split into a training cohort from which the proposed algorithm was derived and a validation cohort on which the model was applied and tested. The training cohort was derived from a random sampling of 80% of the data set, and the validation cohort comprised the remaining 20%. The division process was repeated 1000 times looking for the optimal split that ensures no statistically significant differences between the two cohorts in demographics or AKI outcome. The split was carried on such that the divisions are minimizing the differences of the age, gender, smoking years, and AKI parameters in both the training and validation cohorts. The distribution of the parameters age, smocking, gender, and AKI in both these cohorts are shown in Eq. (1).

1 $$\left[ {\begin{array}{*{20}l} {{\mathbf{Parameter}}} \hfill & {{\mathbf{Training}}\;{\mathbf{Cohort}}} \hfill & {{\mathbf{Validation}}\;{\mathbf{Cohort}}} \hfill \\ {Age} \hfill & {61.23 \pm 12.05} \hfill & {60.92 \pm 13.14} \hfill \\ {Smoking} \hfill & {17.61\% \pm 38.15\% } \hfill & {18.34\% \pm 38.74\% } \hfill \\ {Gender} \hfill & {Male:37.98\% ,Female:62.02\% } \hfill & {35.45\% ,Female:64.55\% } \hfill \\ {AKI} \hfill & {46.84\% \,with\,AKI} \hfill & {38.54\% \,with\,AKI} \hfill \\ \end{array} } \right]$$

### Algorithm

We used the random forest (RF) ML algorithm [22]. We selected the RF algorithm because it can provide a simple explanation of the model’s prediction to healthcare professionals while obtaining a good accuracy on a relatively small data set [23]. We applied the proposed binary AKI prediction decision tree (DT) algorithm on the training cohort and then validated it on the validation cohort that was completed using the sklearn library with Python 3.5. The model’s hyper parameters were determined using the grid search method [24] (see Sect. 2.9) and fivefold cross-validation on the training cohort to determine the values which led to the best performance.

### Feature selection

We performed a feature selection in the following order: first, we manually filtered the features available before the surgery (marked as $F$). Afterward, we evaluated the model’s accuracy, picking one feature from $F$. The feature that resulted in the model’s highest accuracy was chosen ${F}_{1}$. Then, an additional feature from the remaining feature set ($F\backslash {F}_{1}$) was added to the chosen feature set from the previous step such that the model’s accuracy was the highest between all combinations. The process was repeated until the gain in the model’s accuracy upon adding a new feature became less than 1%.

### Model pruning

After training the model, we transformed each DT in the RF into a respective Boolean satisfaction problem (SAT). Each branch was converted into a Boolean condition$r$: ${x}_{1}$ ∧${x}_{2}$∧···∧${x}_{n}$ where ${\left\{{x}_{i}\right\}}_{i=1}^{n}$ are the conditions in each node in the branch and $r$ was the result label node. Branches with the same result label $r$ were stitched together using the’or’ logical operator (∨). Afterward, each Boolean condition was reduced to the minimal Boolean condition that satisfied the same inputs. The result of Boolean condition was converted back into a DT.

### Statistical analysis

We performed a fivefold cross-validation to evaluate the model’s accuracy. The data was divided into five cohorts where four cohorts were used for the training cohort and one for the testing cohort. The process was repeated five times, allowing each patient to be included in both the training and test cohorts. The receiver operating characteristic (ROC) curve was used to measure the model’s classification ability. At each point, the recall and precision were presented in correspondence with a specific decision threshold. The area under the ROC curve (AUC) was used to quantify the model’s classification ability. Finally, the importance of each feature depended on the reduction of classification accuracy caused by removing the feature (e.g., information gain) [25].

### Hyper-parameter fine-tuning

We performed hyper-parameter fine-tuning using the grid search method, based on the model’s accuracy [24]. The grid search was performed on

$${\mathbb{H}}: = [depth,\;MSPL,\;LC,\,n],$$

where depth is an individual DT tree depth; MSPL is the minimal number of samples for a leaf; LC is the leaf count; and n is the number of trees in the RF model.

## Results

### Decision features

Implementation of the method described in Sect. 2.6 on 31 features (see Additional file 1: Appendix) resulted in a set of eight features

2 $$F: = [size,\;renal,\;age,\,baseHB,\,IT,\,weight,\,height,\,creatinine],$$

where size is the size of the tumor in centimeters; renal is the RENAL score; age is the patient’s age in years at the time of the surgery; baseHB is the baseline hemoglobin in g/dL; IT is the ischemia time in minutes; weight is the patient’s weight in kilograms at the time of the surgery; height is the patient’s height in centimeters at the time of the surgery; and creatinine is the baseline pre-operative Creatinine in mg/dL. The model found that IT contributed significantly to the accuracy of the model. However, $IT$ is surgical parameter, and is not available beforehand. In order to overcome this, we defined a feature called $I{T}^{*}$ which is an estimate of the real IT. The $I{T}^{*}$ is obtained using the k-nearest neighbors (KNN) algorithm (where k = 5 and the distance metric is weighted by distance and the average is obtained using the grid search method) on the other seven features which are available before the surgery. To evaluate the quality of the $I{T}^{*}$ feature compared to the original $IT$ feature, we performed a fivefold test on the $IT$ feature with the KNN algorithm. A linear regression on the values ($IT$, $I{T}^{*}$) was obtained, resulting in a coefficient of determination (${R}^{2}$) of 0.879. Namely, the $I{T}^{*}$ feature well estimate the $IT$ feature and therefore, we wereable to replace the feature space to:

3 $$F*: = [size,\;renal,\;age,\,baseHB,\,IT*,\,weight,\,height,\,creatinine].$$

A Pearson correlation coefficient between all pairs of F was then obtained, showing a 0.57 correlation between the renal and size features and 0.45 correlation between patient height and weight. The first correlation is trivial as the renal score includes the size. In addition, the second correlation is already reported in other studies [26]. All other combinations of features from F have absolute correlation below 0.3, supporting the fact that the feature-space is mostly linearly independent.

### AKI classification model

We trained the RF model (see Sect. 2.5) on the clinical data set as described in Sect. 2.4 on the feature set F. Afterward, we performed hyper-parameter fine-tuning as described in Sect. 2.9. Then, we carried out pruning on the best model (see Sect. 2.7). As a result, we obtained a model with 107 DTs; each one of these DTs had up to five levels of depth. The number of leaves is different for each tree in the RF due to the pruning process. The model was validated on the validation set using fivefold cross-validation analysis. The precision obtained 0.69 ± 0.085. Similarly, the recall obtained 0.69 ± 0.062. The features’ importance is presented in Table 1.

**Table 1:** Model’s features importance

Feature	Size	Renal	Age	BaseHB	Weight	Height	Creatinine	IT	IT*
Original importance	0.13	0.11	0.17	0.05	0.15	0.05	0.21	0.13	0
Estimated importance	0.14	0.11	0.19	0.06	0.12	0.03	0.23	0	0.12
Core importance	0.17	0.11	0.2	0.06	0.16	0.06	0.24	0	0

The original importance is the one obtained by a model that uses $IT$. The estimated importance is the one obtained by estimating $IT$ (e.g., $I{T}^{*}$ using the other seven features). The core importance is the importance of the seven features as the weighted average in contribution to the final prediction of $I{T}^{*}$

**Fig. 2:**
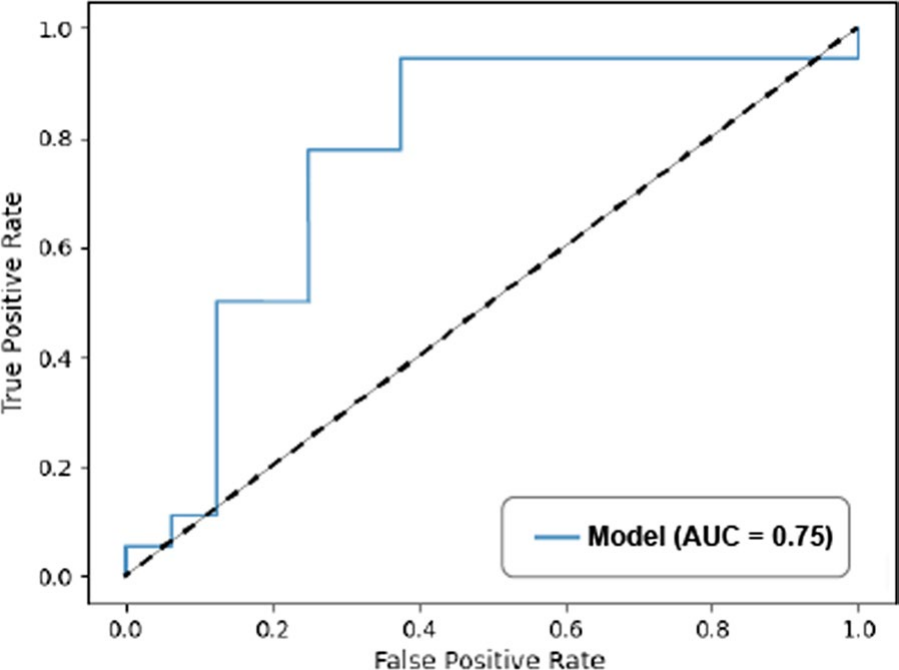
The ROC curve of the model’s prediction on the binary AKI classification, with AUC of 0.75

Furthermore, we derived the ROC curve of the model, as shown in Fig. 2. The AUC was found to be 0.75. In addition, the average confusion matrix was:

4 $$\left[ {\begin{array}{*{20}c} {} & {{\text{True}}} & {{\text{False}}} \\ {{\text{Positive}}} & {0.34} & {0.41} \\ {{\text{Negative}}} & {0.07} & {0.18} \\ \end{array} } \right]$$

### Interface

**Fig. 3:**
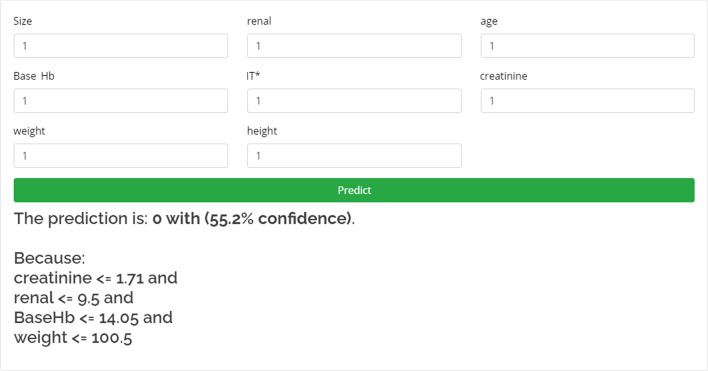
The model’s interface and prediction as a web service. The user inserts a patient’s data into the form and by clicking on the predict button obtains the AKI binary prediction with the model’s confidence. In addition, an explanation of the model’s prediction is provided below

The model has been deployed as a web service.^1^ Figure 3 shows the model’s interface as a web service.

## Discussion

AKI following PN is a unique entity, which significantly differs from medical and post-surgical AKI; in addition to the common risk factors for medical AKI, patients undergoing PN have increased risk for AKI due to the associated blood loss and relative hypovolemia and, more importantly, the clamping of the renal artery and the loss of functional tissue. Several studies reported an increased risk of chronic kidney disease (and mortality) in patients who develop AKI [5, 27–29]. The incidence of AKI following PN varies depending on several parameters, including surgical approach, the definition used for AKI, and the cohort reported in each study. In a recent study by Tachibana et al., the authors reported less than 11% AKI following robotic PN and almost 50%incidence following open PN [30]. Our results demonstrate that the development of AKI following PN can be accurately predicted based only on clinical information routinely collected before surgery. The proposed models performed well according to all evaluation criteria and achieved a higher AUC and accuracy score compared to the classical scoring methods. The proposed model has similar scores to the modern ML-based models [16, 32] and AUC in accordance with previous large studies on general intensive-care unit patients developing medical AKI [31].

Our results agree with a wide range of ML-based AKI risk prediction models from recent years [12, 13]. In particular, our model can be used at any point before the surgery. As such, it is agnostic in time, similar to the approach of several AKI prediction tools [15].

We used a RF model (as an ensemble of DT models) as the ML algorithm for our model in order to take advantage of the explainable property of this model. In addition, by using the SAT pruning algorithm, we were able to obtain the shortest, and therefore, easiest to understand explanation for each prediction. This explanation provides the treating Urologist with the ability to agree or disagree with the model on unique cases and has a second validation process on the model’s prediction based on the personnel’s wider understanding of the patient’s condition (i.e., man-in-the-loop) [33].

The proposed AKI prediction model could be publicly available as an online prognostic calculator, providing a platform for future AKI-prediction studies, and complementing existing risk assessment scores [34]. One could argue that a better endpoint would be the risk of CKD following PN, which is the most important endpoint. In this study, we aimed to predict AKI as it was demonstrated to increase the risk for CKD. A model that predicts CKD is harder to build, and this is one of our future projects.

The main limitation of our study is being an open PN cohort, and it is yet to be determined if it will apply to patients who undergo minimally invasive surgery. The second limitation is the relatively small cohort for this model, although others used smaller cohorts and reported good results [21]. Another limitation is the lack of external validation. Moreover, we used an estimated parameter, ischemia time, to predict the AKI based on pre-operative parameters. However, despite these limitations, our model can predict AKI with relatively high accuracy (75%). In conclusion, our ML model can reliably predict AKI following open PN. Future possible research is to extend the size of the database and perform stability analysis for controversial cases in order to improve the robustness of the proposed model.

## Conclusion

Machine learning algorithms can predict the risk of AKI following open PN.

## Supplementary Information

**Additional file 1.** Raw data.

## Data Availability

All the data that has been used is available in the code repository of the project in Github. Upon acceptance, we will publish all the code used in a GitHub repository. The final outcome can be reviewed at https://teddy4445.github.io/cancer-AKI-predictor-gui/predictor.html.

## Abbreviations

PN: Partial nephrectomy
AKI: Acute kidney injury
CKD: Chronic kidney disease
ML: Machine learning
RF: Random forest
DT: Decision tree
SAT: Boolean satisfaction problem
ROC: Receiver operation characteristic
AUC: Area under the ROC curve
IT: Ischemia time
KNN: K-nearest neighbors

## Footnotes

1 https://teddy4445.github.io/cancer-AKI-predictor-gui/predictor.html.
