# Supplementary material for: Predicting acute kidney injury following open partial nephrectomy treatment using SAT-pruned explainable machine learning model
Source: BMC Med Inform Decis Mak. 2022 May 16;22:133. doi: 10.1186/s12911-022-01877-8 (PMC9112450; doi:10.1186/s12911-022-01877-8)
Supplement: Supplementary file 1 — Additional file 1. Raw data. [file 12911_2022_1877_MOESM1_ESM.docx]

Appendix

Clinical parameters. The data set used in this research is described in Table S1.


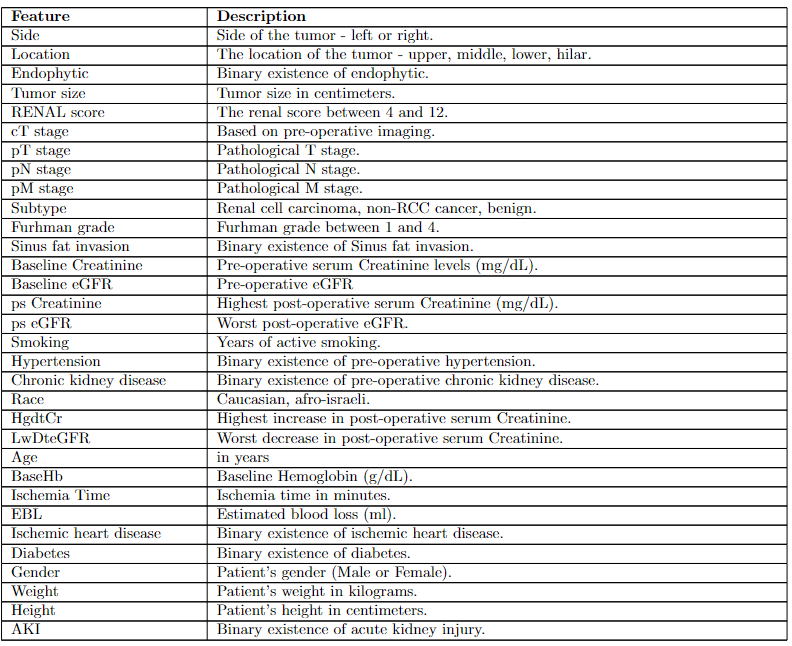


Table S1: Data set feature names and descriptions used in the research

Training-Validation Cohorts Division. The data set is divided into training and validation cohorts with 80% and 20%, respectively. The distribution of the parameters age, smoking, gender, AKI, Baseline creatinine, Baseline GFR, Post operative creatinine, and Post operative creatinine in both these cohorts are shown in Table S2.


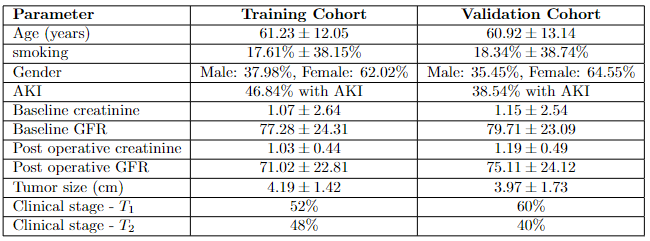


Table S2: The demographical properties of the populations allocated to the training and validation cohorts. The results are shown as mean $\pm$ standard deviation.
